# At the crossroads of religion and palliative care in patients with dementia

**DOI:** 10.1186/s13584-020-00401-5

**Published:** 2020-08-24

**Authors:** Kieran L. Quinn, Daphna L. Grossman

**Affiliations:** 1 Department of Medicine, Divisions of General Internal Medicine and Palliative Care, University of Toronto, Toronto, Canada; 2 Department of Medicine, Sinai Health System and University Health Network, 60 Murray Street, 2nd Floor Room 404, Toronto, Ontario M5T 3L9 Canada; 3 Institute for Health Policy Management and Evaluation, University of Toronto, Toronto, Canada; 4 Department of Family and Community Medicine, North York General Hospital, Toronto, Canada; 5 Department of Family and Community Medicine, Division of Palliative Care, University of Toronto, Toronto, Canada

**Keywords:** Dementia, Palliative care, Judaism

## Abstract

The timing of palliative care initiation may be more appropriately directed using a needs-based approach, instead of a prognostically driven one. Jewish Law or Halachah (“the way”) upholds a strong commitment to the sanctity of life and teaches that the duty to prolong life supersedes the duty to end suffering prematurely, unless one is expected to imminently die. This intersection of palliative care and a reliance on prognostic triggers with an individual’s observance of religious traditions complicates matters nearing the end-of-life. A recent pilot study by Sternberg et al. of 20 patients with advanced dementia in Israel found that home hospice care significantly reduced distressing symptoms, caregiver burden and hospitalization and teaches us important lessons about some of the essential elements to providing excellent palliative care at home, including the 24/7 availability of healthcare providers outside of the emergency department. In light of specific religious practices, palliative care should strive to incorporate a patient’s specific religious observance as part of high-quality end-of-life care.

Palliative care is an approach that focuses on the care of people who are suffering from serious illness with a goal of improving quality of life, reducing suffering and helping with decision making for them and their caregivers [1]. The timing of palliative care initiation may be more appropriately directed using a needs-based approach, instead of a prognostically driven one [2, 3]. In other words, palliative care should be delivered when someone is suffering with a serious illness and in need of care to relieve that suffering. It is easy to imagine that this may occur at any point along the disease trajectory independent of the timing of their death, including at the time of diagnosis [4].

However, the provision of palliative care services is often determined in the context of prognosis, with enhanced services offered as a person approaches end-of-life. “Trigger tools” or prognostication calculators have been developed to answer the question of how soon a person will die and therefore signal when palliative care should be instituted. There is increasing interest in the use of machine learning and artificial intelligence to more accurately determine a person’s prognosis [5, 6]. However, pinning our hopes on complex machines to predict complex events such as the timing of a person’s death in order to more accurately determine the timing of the provision of palliative care services, may be misleading.

On the surface, the logic is sound. If medicine can more accurately predict the timing of a person’s death, then the timing for providing palliative care should be obvious. This may be especially applicable to chronic non-cancer illnesses such as dementia, where there is currently less prognostic accuracy [7, 8]. Yet the intersection of our reliance on prognostic triggers with an individual’s observance of religious traditions can further complicate matters nearing the end-of-life. Jewish Law or Halachah (“the way”) upholds a strong commitment to the sanctity of life [9, 10]. It teaches that the duty to prolong life supersedes the duty to end suffering prematurely, unless one is deemed to be a goses and expected to imminently die (although there is currently no accepted consensus as to the timing) where interference with the dying process is prohibited [9, 10]. In 2005, the Israeli Parliament passed a law regulating the treatment of dying patients to provide legal guidance surrounding the bioethical dilemmas that arose related due to its cultural and religious diversity. It defined a “terminally ill patient” as one who will die within 6 months and a “terminally ill patient in the final stages of life” as one who will die within 2 weeks, even if appropriate therapy is given [11]. However, ascertaining the exact timing as to when a person would be in the final stages of life is difficult because the exact timing of death cannot be accurately predicted. If clinical decisions are made strictly in the context of one’s duty to observe Halacha, then concepts about care such as “not adding to quality of life” or “may likely have little benefit” (like feeding tubes or artificial means of hydration in patients with advanced dementia) will be irrelevant to that decision making.

An understanding of Jewish bioethics and religious diversity is paramount to placing in context a recent pilot study by Sternberg et al. of home hospice care for 20 patients with advanced dementia who were cared for under the Maccabi Healthcare Services, the second largest HMO in Israel [12]. The authors employed the use of a multidisciplinary team who visited the patients in their homes and were available 24/7 to provide support during moments of crisis. Study participants were eligible if they had severe dementia, defined as stage 7 or higher on the Global Deterioration Scale (GDS). The potential for suffering was palpable: patients scoring ≥7 on the GDS were profoundly cognitively impaired (inability to recognize and communicate with family members), were unable to independently ambulate, and had complete functional dependence for their activities of daily living including incontinence of urine and stool. These patients had a high burden of medical comorbidity and had spent an average of 2 weeks in hospital within the past year.

The intervention was a resounding success. Patients and their families were supported by the program for up to 6 months, the equivalent of the hospice benefit provided under Medicare in the United States. Home-hospice care for patients with advanced dementia significantly reduced distressing symptoms, caregiver burden and hospitalization. Caregivers were especially appreciative of the 24/7 availability of healthcare providers (25% of calls were made outside regular working hours) which contributed to high overall satisfaction with their care.

How should we interpret and apply these important findings in the context of religious practice? First, an important minority of Israelis are observant of Halachah. The majority of Halakhic opinion is also not opposed to treating suffering (even at the risk of shortening life) if the primary intent is to reduce suffering such as when treating pain with opioids. Therefore, it is essential that all palliative care clinicians be educated in and inquire about their patients’ specific religious and spiritual practices to provide patient-centred care. Second, 20% of patients were fed artificially, a smaller percentage than one would expect in Israel, where artificial nutrition approaches 53%, and lower than the approximately one third of residents in nursing homes in the United States with severe cognitive impairment who have feeding tubes [13, 14]. Such a stark contrast may indicate that patients and their families in this study were more oriented and accepting of a palliative approach to care. This may represent a form of selection bias, but the results are still helpful to a large number of people as palliative care should not be viewed as a “one size fits all” intervention. Further, supporting patient goals-directed care is essential to providing high quality end-of-life care [2], which should strive to incorporate one’s specific religious observance as part of that care. Palliative care clinicians supporting the minority of patients and their caregivers in Israel who abide by Halachah need to understand the importance of the duty to prolong life and that it supersedes the duty to end suffering prematurely – unless they can accurately predict that their patient is terminally ill and in the final stages of life. These competing priorities may feel uncomfortable to the palliative care clinician who traditionally focusses on quality over quantity of life, but are paramount to high quality end of life care for some. Third, 65% of patients in the study survived beyond 6 months and returned to their usual homecare program. Under Israeli Law, these patients would not necessarily be considered terminally ill, although this can only be determined after the fact, and further highlights medicine’s inability to accurately predict survival. Healthcare systems should look to improve care by expanding access to home-based palliative care throughout the patient’s entire illness trajectory [15], and not limit it to a specific time-frame near the end of life; this is especially true when our ability to predict that timing is only modest at best. Finally, to meet the growing demand for palliative care, it is time to make targeted investments in developing new models of home care and training all clinicians to provide palliative care to older adults with serious illness. The study by Sternberg et al. teaches us important lessons about some of the essential elements to providing excellent palliative care at home, including the 24/7 availability of healthcare providers outside of the emergency department.

Dementia is but one disease among many that require creative and novel solutions such as home hospice to ensure the delivery of high-quality palliative care to all patients with serious illness. When at the crossroads of religion and palliative care, a paraphrasing of the fabled Blues musicians Robert Johnson and Eric Clapton teaches us that home hospice is a place where everyone seems to know you and no one passes you by [16].
